# Ultrasound-Guided Ilioinguinal/Iliohypogastric Block Mitigates Catheter-Related Bladder Discomfort After Prostate Surgery: A Prospective Randomized Controlled Trial

**DOI:** 10.7759/cureus.94313

**Published:** 2025-10-10

**Authors:** Jing Zhang, Shan Song

**Affiliations:** 1 Anaesthesiology, Yantai Yuhuangding Hospital, Shandong, CHN; 2 Anesthesiology, The Affiliated Yantai Yuhuangding Hospital of Qingdao University, Shandong, CHN

**Keywords:** catheter-related bladder discomfort, elderly, iliohypogastric-ilioinguinal nerve block, postoperative complications, transurethral resection of prostate, ultrasound guidance

## Abstract

Background

Catheter-related bladder discomfort (CRBD) is a common and distressing complication of transurethral resection of the prostate (TURP), especially in elderly patients. However, effective prevention strategies are lacking. This study aimed to evaluate the efficacy of ultrasound-guided iliohypogastric-ilioinguinal nerve block (IINB) in alleviating CRBD.

Methods

Eighty elderly male patients who underwent TURP were randomly assigned to the IINB group (n = 40), which received bilateral iliosubcostal-ilioinguinal nerve blocks after general anesthesia with 15 ml of 0.33% ropivacaine on each side, or the control group (n=40), which received only standard general anesthesia. The primary outcome was the severity of CRBD (rated on a 0-3 scale) at 5 min, 1 h, 3 h, 6 h, 12 h, and 24 h postoperatively. Secondary outcomes included postoperative pain visual analog scale (VAS) scores, intraoperative opioid consumption, post-anesthesia care unit (PACU) stay duration, and other postoperative complications.

Results

This study evaluated the impact of nerve block (Group N) versus control (Group C) on CRBD, pain scores, opioid consumption, and recovery parameters in patients undergoing TURP.

Group N exhibited significantly lower CRBD scores at 5 min (p<0.001), 1 h (p<0.001), 3 h (p<0.001), and 6 h (p<0.05) postoperatively, with no differences at 12 h or 24 h. Similarly, VAS pain scores were reduced in Group N at all early timepoints (5 min to 6 h; p<0.05 to p<0.001), aligning with the CRBD findings. Intraoperatively, Group N required less sufentanil (26.25 ± 5.63 μg vs. 32.88 ± 6.29 μg; p<0.001) and remifentanil (170.45 ± 53.63 μg vs. 217.88 ± 67.79 μg; p=0.001), and demonstrated a shorter extubation time (6.83 ± 1.75 min vs. 8.13 ± 1.49 min; p=0.001). PACU stay duration did not differ significantly (p=0.309). The incidence of postoperative complications, including postoperative nausea and vomiting (PONV), chills, hypoxemia, and sinus bradycardia, showed no statistically significant differences between groups (all p>0.05), as analyzed by Fisher’s exact test.

## Introduction

Catheter-related bladder discomfort (CRBD), characterized by an urgent desire to urinate accompanied by discomfort, pain, and behavioral responses, is a frequent complication of urinary catheterization, particularly after transurethral resection of the prostate (TURP) [1]. Its incidence is reported to be as high as 55% in general surgical patients and can exceed 90% in urological procedures, severely affecting patient satisfaction and the quality of early recovery [2,3].

The pathophysiology of CRBD is primarily attributed to involuntary contractions of the detrusor muscle, mediated by muscarinic receptors stimulated by an indwelling catheter [4]. Conventional treatments, including anticholinergic agents (e.g., tolterodine and oxybutynin) and analgesics (e.g., gabapentinoids), often provide suboptimal relief and are associated with undesirable side effects, such as dry mouth, sedation, dizziness, and cognitive dysfunction [5,6]. These side effects are particularly concerning in elderly patients undergoing TURP, who demonstrate increased sensitivity to medication side effects and are more vulnerable to both CRBD and its pharmacological management [7].

Regional anesthesia techniques offer a promising alternative by targeting the somatic afferent innervation of the bladder. The iliohypogastric and ilioinguinal nerves (T12-L1) provide sensory innervation to the suprapubic region and bladder dome [8]. Ultrasound-guided regional anesthesia has gained prominence due to its enhanced safety profile, with large-scale analyses demonstrating low complication rates when proper protocols are followed [9,10]. Specifically, ultrasound-guided iliohypogastric-ilioinguinal nerve block (IINB) is a well-established technique for providing analgesia in inguinal surgery, with expanding applications in urological procedures [11-12]. The favorable pharmacokinetic profile of local anesthetics, such as ropivacaine, supports their use in providing sustained postoperative analgesia [13].

Therefore, it is of great clinical significance to improve the existing operation techniques and adopt a relatively easy-to-perform, safe, and reliable ultrasound-guided iliohypogastric and ilioinguinal nerve block method that can overcome the above adverse effects. In clinical practice, we found that placing the ultrasound probe along the long axis of the inguinal region and inserting the needle from above the inguinal region, and implementing the iliohypogastric and ilioinguinal nerve block using the in-plane technique, is more convenient in operation and can effectively reduce the risk of puncture needle injury to abdominal organs and avoid other disadvantages [14].

We hypothesized that ultrasound-guided IINB would reduce the incidence and severity of CRBD in elderly patients after TURP. This prospective, randomized, controlled study aimed to test this hypothesis and evaluate the impact of the block on opioid consumption and postoperative.

## Materials and methods

Patient population

Eighty patients with American Society of Anesthesiologists (ASA) physical status II-III, aged 60-80 years, scheduled for elective TURP under general anesthesia were enrolled. Exclusion criteria included: patient refusal; body mass index >35 kg/m², <18 kg/m²; pre-existing neuropathic pain or chronic opioid use; coagulopathy; infection at the injection site; known allergy to local anesthetics; pre-existing urinary incontinence or neurological bladder dysfunction; and severe hepatic or renal impairment.

Randomization and blinding

Patients were randomly allocated to either the control group (C group) or the IINB group (N group) using computer-generated random number sequences concealed in opaque envelopes. An anesthesiologist who was not involved in patient assessment or data collection performed randomization and nerve blocks. Patients, surgeons, and anesthesiologists who collected the postoperative data were blinded to the group assignment.

Measurements and outcomes

The primary outcome was the severity of CRBD, assessed on a 4-point scale (0, no symptoms; 1, mild, reported only on questioning; 2, moderate, reported without questioning but without behavioral response; 3: severe, reported with behavioral response such as flailing limbs or attempts to pull out the catheter) at 0 (on arrival in the post-anesthesia care unit (PACU)), 0.5, 3, 6, 12, and 24 hours postoperatively.

The secondary outcomes included the following: Postoperative pain intensity was assessed using a visual analog scale (VAS, 0-10) at the same time points, intraoperative consumption of sufentanil and remifentanil, duration of stay in the PACU, occurrence of adverse events (e.g., nausea, vomiting, and block-related complications).

Statistical analysis

Based on a pilot study, a sample size of 36 patients per group was required to detect a 1-point difference in the CRBD score (standard deviation (SD)=1.2), with 90% power and an alpha error of 0.05. We enrolled 40 patients per group to account for potential dropout. The data were analyzed using SPSS 25.0. Normality was assessed using the Shapiro-Wilk test. Continuous normally distributed data are presented as mean ± SD and compared using the independent samples t-test. Non-normally distributed data (CRBD scores and VAS scores) are presented as median (interquartile range) and compared using the Mann-Whitney U test. Categorical data are presented as numbers (%) and were compared using the Chi-square or Fisher's exact test. Repeated-measures Analysis of Variance (ANOVA) was used to analyze changes over time. Statistical significance was defined as a two-tailed P-value of < 0.05.

## Results

**Figure 1:**
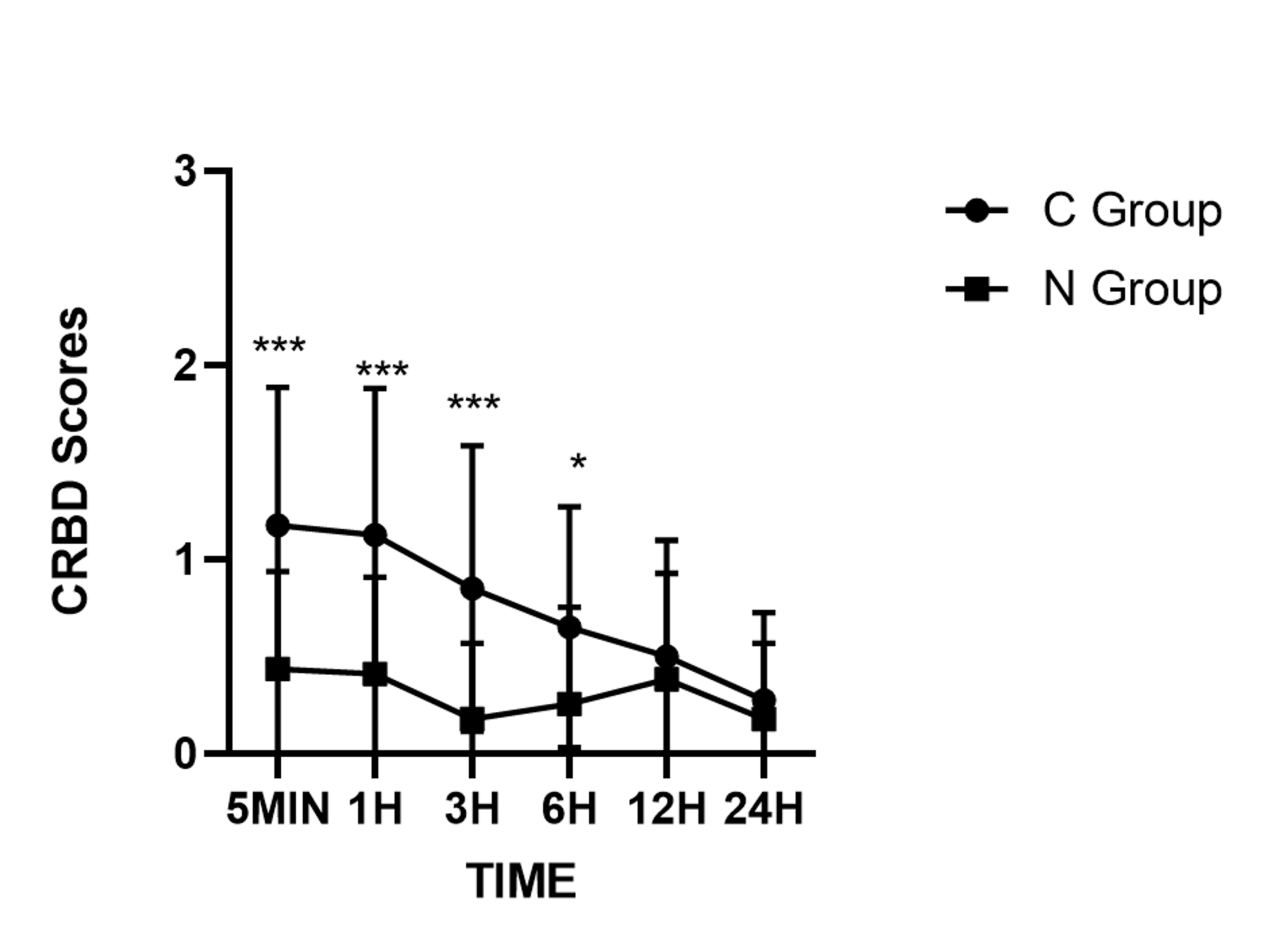
CRBD scores in two groups at different time points Comparison of CRBD scores between the C Group and the N Group at different time points. Data are presented as mean ± Standard Error of the Mean (n = 40 per group). Error bars represent the Standard Error of the Mean. An ANOVA model is a statistical method that partitions the total variance in a dataset into different sources, typically to test if there are statistically significant differences between the means of three or more groups. Statistical significance was determined by two-way repeated-measures ANOVA followed by Bonferroni’s post hoc test. CRBD: catheter-related bladder discomfort *P < 0.05, **P < 0.01, ***P < 0.001 vs. C Group at the corresponding time point.

To assess the impact of nerve block on CRBD following TURP, we used a line chart to compare the CRBD scores between the nerve block group (N Group) and the control group (C Group) at various postoperative intervals (Figure 1). Our analysis revealed that the N Group exhibited significantly reduced CRBD scores relative to the C Group at 5 min, 1 h, 3 h, and 6 h (P < 0.05, P < 0.001, respectively), demonstrating the efficacy of nerve blockade in mitigating early CRBD. In contrast, no statistically significant differences were detected at 12 and 24 h, implying time-dependent attenuation of this therapeutic benefit.

To assess the impact of nerve block on CRBD following TURP, we used a line chart to compare the CRBD scores between the nerve block group (N Group) and the control group (C Group) at various postoperative intervals (Figure 1). Our analysis revealed that the N Group exhibited significantly reduced CRBD scores relative to the C Group at 5 min, 1 h, 3 h, and 6 h (P < 0.05, P < 0.001, respectively), demonstrating the efficacy of nerve blockade in mitigating early CRBD. In contrast, no statistically significant differences were detected at 12 and 24 h, implying time-dependent attenuation of this therapeutic benefit.

Secondary outcomes

VAS Scores

**Figure 2:**
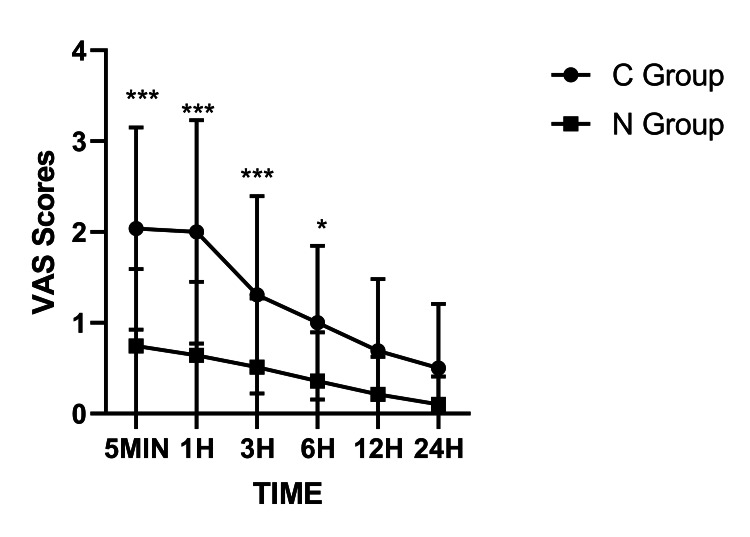
VAS scores in two groups at different time points Data are presented as mean ± SEM (n = 40 per group). Error bars represent the SEM derived from the repeated-measures ANOVA model. Statistical significance was determined by two-way repeated-measures ANOVA followed by Bonferroni’s post hoc test. *P < 0.05, **P < 0.01, ***P < 0.001 vs. C Group at the corresponding time point. VAS: visual analog scale; SEM: standard error of the mean

To assess the efficacy of IINB in managing pain following TURP, we analyzed VAS scores across six postoperative time points using two-way repeated-measures ANOVA with Bonferroni correction (Figure 2). The nerve block group (N Group) displayed significantly reduced pain scores compared to the control group (C Group) at all intervals from 5 min to 24 h (P < 0.05, P < 0.001), with the most substantial differences evident within the first 6 h. Although both groups exhibited a gradual decline in pain over time, the N Group consistently sustained near-minimal VAS scores throughout the observation period. These results demonstrated that the nerve block technique offers effective and prolonged postoperative analgesia.

Opioid differences and quality of awakening

To evaluate differences in opioid utilization and recovery outcomes, we compared analgesic dosing and perioperative metrics between the C (n = 40) and N (n = 40) groups (Table 1). Our analysis revealed that the N group demonstrated significantly reduced sufentanil consumption (26.25 ± 5.63 μg versus 32.88 ± 6.29 μg) and lower remifentanil requirements (170.45 ± 53.63 μg versus 217.88 ± 67.79 μg) relative to the C group. Furthermore, extubation time was shorter in the N group (6.83 ± 1.75 min) than in the C group (8.13 ± 1.49 min). In contrast, PACU stay duration did not differ significantly between groups (35.45 ± 6.95 min vs. 37.13 ± 7.67 min). These results indicate that the N protocol may minimize opioid use and facilitate quicker emergence from anesthesia.

**Table 1:** Comparison of intraoperative opioid consumption and postoperative recovery quality between groups *p<0.05 **p<0.01

	C Group (n=40)	N Group (n=40)	t-value	p-value
Sufentanil (μg)	32.88±6.29	26.25±5.63	4.961	0.000**
Refentanil (μg)	217.88±67.79	170.45±53.63	3.470	0.001**
Extubation Time (min)	8.13±1.49	6.83±1.75	3.576	0.001**
Post-anesthesia care unit (PACU) stay time) min)	37.13±7.67	35.45±6.95	1.023	0.309

Postoperative complications

As the expected frequency is less than 5, Fisher’s exact test is more appropriate for the statistical analysis of postoperative complications (Table 2). To evaluate perioperative complication rates between groups C and N, we conducted a statistical analysis of four adverse events: nausea and vomiting, chills, hypoxemia, and sinus bradycardia. The results demonstrated incidence rates of 15% versus 7.5% (p=0.48) for nausea and vomiting, 10% versus 5% (p=0.68) for chills, 5% versus 2.5% (p>0.99) for hypoxemia, and 5% versus 0% (p=0.49) for sinus bradycardia in groups C and N, respectively. As all p-values were greater than 0.05, the findings suggest no statistically significant differences in complication incidence between the groups.

**Table 2:** Comparison of intraoperative and Postoperative complications between groups The results demonstrated incidence rates of 15% versus 7.5% for nausea and vomiting, which corresponded to a risk difference of 7.5% (95% CI: -5.3 to 20.3; p=0.48). As all p-values were greater than 0.05 and all confidence intervals for the effect sizes crossed zero (for RD) or one (for OR), the findings suggest no statistically significant differences in complication incidence between the groups.

Complication	C Group (n=40)	N Group (n=40)	Statistical Value	Effect Size (RD/OR)	95% Confidence Interval	p-value
Nausea and vomiting	15%	7.5%	Fisher’s exact	RD=7.5% OR=2.18	RD: -5.3% to 20.3%; OR: 0.43 to 11.15	0.48
Chills	10%	5%	Fisher’s exact	RD=5% OR=2.11	RD: -6.3% to 16.3%; OR: 0.33 to 13.70	0.68
Hypoxemia	5%	2.5%	Fisher’s exact	RD=2.5% OR=2.05	RD: -5.4% to 10.4%; OR: 0.18 to 23.90	>0.99
Sinus bradycardia	5%	0%	Fisher’s exact	RD=5%	RD: -2.9% to 12.9%	0.49

## Discussion

Our study demonstrates that preoperative ultrasound-guided IINB significantly reduces the severity of CRBD in elderly patients undergoing TURP. The intervention not only alleviated CRBD during the first 12 postoperative hours but also decreased intraoperative opioid requirements and improved postoperative recovery quality, without increasing complications.

Mechanistic basis and comparative efficacy with regional techniques

The observed reduction in CRBD severity finds support in the anatomical understanding of bladder sensory innervation [8] and the established pathophysiology of CRBD [4]. The bladder dome receives somatic sensory input through the iliohypogastric and ilioinguinal nerves (T12-L1) [8], and our results substantiate the hypothesis that blocking these pathways effectively mitigates afferent signals generated by catheter-induced bladder irritation. This mechanism differs fundamentally from previously studied approaches targeting the pudendal nerve or dorsal penile nerve [15,16], which primarily address perineal and penile discomfort. While these alternative regional techniques have demonstrated efficacy in reducing CRBD [15,16], our IINB technique provides a more direct approach to mitigating bladder dome irritation.

The opioid-sparing effect observed in our study carries particular clinical significance for elderly surgical patients. This population demonstrates increased sensitivity to opioid-related adverse effects, including respiratory depression, postoperative cognitive dysfunction, and delirium [17,18]. Our findings corroborate previous work demonstrating that regional anesthesia techniques can significantly reduce perioperative opioid consumption while maintaining adequate analgesia [19]. The favorable safety profile of ultrasound-guided regional anesthesia techniques [9,10] further supports the clinical utility of IINB in this vulnerable population.

Advantages over pharmacological approaches

When compared to pharmacological strategies for CRBD management, our IINB technique offers several distinct advantages. While anticholinergic agents, gabapentinoids, and other systemic medications have demonstrated efficacy, they often produce side effects-including cognitive impairment, sedation, and cardiovascular effects-that are particularly problematic in elderly patients [5,6,20]. The targeted nature of IINB minimizes systemic exposure and associated adverse events. The temporal pattern of CRBD reduction in our study (significant effects up to 6 hours postoperatively) corresponds with the expected duration of action for ropivacaine nerve blocks and provides more sustained relief than single-dose pharmacological interventions [13].

Safety profile and technical considerations

The safety profile of ultrasound-guided IINB in our study appears favorable, with no block-related complications observed. This aligns with established literature on ultrasound-guided abdominal wall blocks and large-scale registry data demonstrating the safety of ultrasound-guided nerve blocks [9-11]. The real-time visualization afforded by ultrasound allows for precise needle placement and injection, thereby minimizing risks of visceral injury and intravascular injection [9,10]. However, it is imperative to acknowledge that our study was not specifically powered to detect rare adverse events, and the absence of a standardized screening protocol for subtle complications represents a limitation.

Clinical implications and limitations

Our findings contribute to the growing body of evidence supporting regional anesthesia techniques for CRBD management within enhanced recovery after surgery protocols [14]. The expanding applications of regional anesthesia techniques in urological procedures have been well documented, with increasing evidence supporting their safety in elderly populations [12]. However, our study has several limitations that should be acknowledged. First, although we reported CRBD incidence, we did not systematically assess other important patient-centered outcomes such as patient satisfaction scores, detailed duration of individual CRBD episodes, or a comprehensive set of block-specific complications. This limitation affects the comprehensiveness of our safety and efficacy assessment. Additionally, the relatively small sample size (n=80) may have limited our ability to detect rare complications or subtle differences in secondary outcomes. The single-center design and homogeneous patient population may also affect the generalizability of our findings.

Given these limitations, particularly the non-assessment of patient satisfaction, detailed CRBD duration, and broader complication profiles, our findings should be interpreted with caution. Future multicenter studies with larger cohorts and more comprehensive outcome assessments are needed to confirm these results and explore potential variations in efficacy across different patient subgroups.

## Conclusions

Our study demonstrates that although preoperative ultrasound-guided IINB significantly alleviates CRBD, reduces opioid consumption, and enhances recovery within the first 6 hours postoperatively, the transient nature of this benefit and the omission of key outcomes like patient satisfaction and a full safety profile mean that these findings must be interpreted with caution. Consequently, while promising, the integration of IINB into multimodal analgesic protocols for elderly TURP patients should be considered preliminary and awaits confirmation from future studies designed with broader outcome measures.
